# Protective Effects of Bacteriocin-Producing *Lactiplantibacillus plantarum* on Intestinal Barrier of Mice

**DOI:** 10.3390/nu15163518

**Published:** 2023-08-10

**Authors:** Yushan Bu, Yisuo Liu, Yinxue Liu, Jiayuan Cao, Zhe Zhang, Huaxi Yi

**Affiliations:** 1 College of Food Science and Engineering, Ocean University of China, Qingdao 266000, China; buyushan@stu.ouc.edu.cn (Y.B.); liuyisuo@stu.ouc.edu.cn (Y.L.); jiayuanalice@163.com (J.C.); 2 Food Laboratory of Zhongyuan, Luohe 462300, China

**Keywords:** bacteriocin, *Lactiplantibacillus plantarum*, intestinal barrier

## Abstract

Bacteriocins are crucial metabolites of probiotics that display beneficial functions. The intestinal barrier is an important target on which probiotics exert their intestinal health activity. However, the impacts of bacteriocin-producing probiotics on the intestinal barrier are unclear. In this study, the effects of bacteriocin-producing *Lactiplantibacillus plantarum* Q7 and *L. plantarum* F3-2 on the intestinal barrier of mice were explored. It was shown that *L. plantarum* Q7 promoted the expression of mucin MUC2 to enhance the protection provided by the intestinal mucus layer. *L. plantarum* Q7 up-regulated the gene expression of intestinal tight junction proteins *ZO-1* and *JAM-1* significantly, and *L. plantarum* F3-2 up-regulated *ZO-1* and *Claudin-1* markedly, which exhibited tight junction intestinal barrier function. The two strains promoted the release of IgA and IgG at varying degrees. The antimicrobial peptide gene *RegIIIγ* was up-regulated markedly, and the gene expression of inflammatory cytokines appeared to exhibit an upward trend with *L. plantarum* Q7 treatment, so as to enhance intestinal immune regulation function. Furthermore, *L. plantarum* Q7 and *L. plantarum* F3-2 increased the abundance of the beneficial bacteria *Muribaculaceae*, inhibited the growth of the harmful bacteria *Parabacteroides*, and facilitated the synthesis of total short-chain fatty acids (SCFAs), which seemed to favor the prevention of metabolic diseases. Our results suggested that *L. plantarum* Q7 and *L. plantarum* F3-2 showed strain specificity in their protective effects on the intestinal chemical, physical, immunological and biological barriers of mice, which provided theoretical support for the selective utilization of bacteriocin-producing strains to regulate host health.

## 1. Introduction

Probiotics have been confirmed as active microorganisms that play advantageous roles in the body when adequate amounts are ingested [1]. In recent years, a variety of beneficial functions of probiotics have been continuously proven, which are implicated in secreting antibacterial factors, modulating the immune system, enhancing bowel motility and protecting the intestinal barrier [2]. The intestinal barrier is the main place in which the prevention of organism infection and inflammation occurs, and is the first line of defense against pathogens invasion [3]. It is composed of chemical, physical, immunological and biological barriers, which can help ensure the maximum intake and utilization of nutrients, and plays the role of preventing pathogenic bacteria and harmful substances from entering the body [4,5]. With the increasing researches on probiotics and intestinal health, several functions of probiotics have been proven to be correlated with their role of regulating the intestinal barrier [6].

The intestinal chemical barrier is mainly formed of the mucus layer. When the intestinal epithelium is invaded by pathogenic microorganisms, some probiotics can induce epithelial cells to secrete mucins that reduce the binding of intestinal pathogens to mucosal epithelial cells, which strengthens the intestinal mucosal barrier’s function [7]. As an important defense line between the intestinal cavity and internal environment, the intestinal physical barrier mainly consists of enterocytes and tight junction complexes, and plays a crucial role in protecting the intestinal tract and body health [8]. Han et al. [9] pointed out that *L. plantarum* GL17 and *Lactiplantibacillus brevis* AY858 enhanced the expression of tight junction proteins such as *ZO-1*, *Occluding* and *Claudin-1* to improve the integrity of the gut barrier and protect mice from intestinal inflammation. The intestinal immunological barrier is essential for appropriate immune defense and inflammation control. Probiotics can stimulate the anti-inflammatory or pro-inflammatory immune response in the intestine to enhance immunity function [10], and they also alleviate inflammation by inhibiting pro-inflammatory factors to treat inflammatory bowel disease (IBD) [11]. Intestinal microorganisms are the important part of the intestinal biological barrier, and are regarded as vital target on which probiotics to exert their beneficial functions [12]. It was shown that *L. plantarum* HAC01 administration regulated the intestinal microbiota, thereby ameliorating metabolic syndrome in mice [13].

Bacteriocins are important metabolites produced by probiotics and exhibit biological antibacterial, anti-infection, anti-inflammation and immunomodulation activities [14]. The main contributions of bacteriocins toward probiotics exerting their functions in the intestine include inhibiting pathogenic microorganisms growth, promoting the survival and colonization of probiotics in the gut, regulating the balance of the intestinal microbiota and alleviating the inflammation response [15,16,17]. Although bacteriocin-producing probiotics have been confirmed to show probiotic effects on the intestinal health of their host, current researches are mostly focused on alleviating intestinal pathogens infection and regulating the gut microbiota structure, and there is still a lack of systematic study on their influences on the intestinal barrier. Consequently, it is of great importance to explore the functional impacts of bacteriocin-producing probiotics on the intestinal barrier for the utilization of probiotics to improve host gut health.

Our preliminary research showed that *L. plantarum* Q7 and *L. plantarum* F3-2 have been proven to exhibit a bacteriocin-producing capacity and probiotic potential *in vitro* [18]. In this study, we aimed to investigate the effects of *L. plantarum* Q7 and *L. plantarum* F3-2 on the intestinal chemical, physical, immunological and biological barriers *in vivo*, which would provide a scientific basis for the development of safe and effective bacteriocin-producing probiotics.

## 2. Materials and Methods

### 2.1. Strain Cultivation and Bacterial Suspension Preparation

*L. plantarum* Q7 and *L. plantarum* F3-2 were isolated from yak milk and infant feces, respectively. The two strains were cultured in de Man, Rogosa, and Sharpe (MRS) broth (Hopebio Technology, Qingdao, China) at a 2% (*v*/*v*) inoculation amount and activated twice at 37 °C for 24 h. The bacterial cells were collected via centrifugation (6000 rpm, 10 min, 4 °C) and washed twice with phosphate-buffered saline (PBS), after which deposits were dissolved in PBS, and the concentration was 1 × 10^9^ CFU/mL.

### 2.2. Animals and Treatments

Specific pathogen-free (SPF) C57BL/6J mice (male, 6 weeks old) were used in the experiment. All mice were kept in an animal care facility (22 ± 2 °C temperature, 50 ± 10% humidity, 12-h light/dark cycle) and sufficient food and water were provided. Mice were acclimated to the environment for 7 days, and then randomly assigned to 3 groups (n = 12 in each group): the control group (control), which was treated with 200 μL PBS, and the L. plantarum Q7 group (Q7) and L. plantarum F3-2 group (F3-2), which were treated with 200 μL L. plantarum Q7 or L. plantarum F3-2 suspension, respectively. The total oral administration duration was 4 weeks. Mice were weighed weekly, and food and water consumption were recorded every 3 days. Fecal samples were collected from each mouse on the last day of oral gavage. After mice were anesthetized and euthanized, eyeball blood was extracted, which was allowed to stand for 2 h, and then centrifuged (4000 rpm, 40 min, 4 °C) to obtain serum. All experiments were approved and supervised by Animal Ethics Committee of Ocean University of China (permission number: SPXY2022030802).

### 2.3. Histomorphological and Immunohistochemical Evaluation

After the ileal and colonic tissues were fixed in 4% paraformaldehyde (Servicebio Technology, Wuhan, China) and dehydrated, the paraffin-embedded sections were dewaxed, stained with hematoxylin and eosin (H&E) (Servicebio Technology, Wuhan, China), and visualized using a microscope (Nikon, Tokyo, Japan). For the immunohistochemical evaluation of mucin MUC2 expression, the colonic sections were subjected to antigen repair, and 3% hydrogen peroxide solution (Sinopharm, Beijing, China) was added to block endogenous peroxidase activity. The tissues were sealed with 3% BSA (Servicebio Technology, Wuhan, China) at room temperature for 30 min, and then incubated with a primary antibody overnight at 4 °C. After incubation with a secondary antibody, the sections were stained with DAB (Servicebio Technology, Wuhan, China) and hematoxylin, and observed under a microscope.

### 2.4. Enzyme-Linked Immunosorbent Assay (ELISA)

The ileum and colon were homogenized in RIPA lysis buffer (Beyotime Biotechnology, Shanghai, China) using a tissue grinder (Servicebio Technology, Wuhan, China) prior to centrifugation (3000 rpm, 20 min, 4 °C). The supernatants were obtained and the levels of IgA and IgG in the serum, ileum and colon were measured following the directions of the ELISA kit (CalvinBio, Suzhou, China).

### 2.5. Real-Time Quantitative Polymerase Chain Reaction (RT-qPCR) Analysis

Total RNA was extracted from the ileal and colonic tissues with TRNzol Universal (Tiangen, Beijing, China), and cDNA was synthesized using ReverTra Ace qPCR RT Master Mix with gDNA Remover (Toyobo, Osaka, Japan). Genes were quantitated on a CFX96 Real-Time PCR System (Bio-rad, Hercules, CA, USA) in a volume of 25 μL. β-actin was used as a housekeeping gene and the 2^−ΔΔCt^ method was utilized to calculate the relative expression of genes. The specific primers for RT-qPCR are listed in Table 1.

### 2.6. 16S rRNA Gene Sequencing

The extracted genomic DNA samples from mice feces were used as the template to amplify the V3-V4 regions of bacterial 16S rRNA genes using the forward primer 338F (ACTCCTACGGGAGGCAGCA) and reverse primer 806R (GGACTACHVGGGTWTCTAAT). The libraries were built using TruSeq Nano DNA LT Library Prep Kit (Illumina, San Diego, CA, USA), and qualified libraries were underwent paired-end 2 × 250 bp sequencing on an Illumina NovaSeq platform. The high-quality sequences were assigned to OTUs with 97% clustering thresholds by UCLUST. The sequence data were analyzed using QIIME2 2019.4 software and the R package.

### 2.7. SCFAs Quantification

SCFAs in fecal samples were determined via gas chromatography–mass spectrometry (Agilent Technologies, Palo Alto, CA, USA) using the method of Han et al. [19] with some modifications. About 0.1 g of fecal samples were homogenized in 600 μL ultrapure water and acidified using 50% concentrated sulfuric acid (Sinopharm, Beijing, China) prior to centrifugation (5000× *g*, 10 min, 4 °C). The supernatants were taken and vortexed with anhydrous ether (Sinopharm, Beijing, China) at 1: 1 (*v*/*v*), after which the samples were centrifuged (5000× *g*, 10 min, 4 °C), and the ether layer was collected for analysis. SCFAs were separated using an HP-FFAP column (30 m × 250 μm × 0.25 μm; Agilent Technologies, Palo Alto, CA, USA). The initial temperature was 90 °C, which was elevated to 150 °C at 12 °C/min. Then, the temperature was elevated to 220 °C at 20 °C/min and held for 4.5 min. Acetic acid, propionic acid and butyric acid (Macklin Biochemical, Shanghai, China) were the standard solutions.

### 2.8. Statistical Analysis

All values were expressed as mean ± SD. An independent-samples *t*-test was used for comparing statistical differences between two groups, and multiple comparisons were evaluated by ANOVA and Duncan’s test using IBM SPSS Statistics 22.0. The criterion for significance was *p* < 0.05.

## 3. Results

### 3.1. Effects of Bacteriocin-Producing L. plantarum on Physiological Indexes of Mice

**Figure 1:**
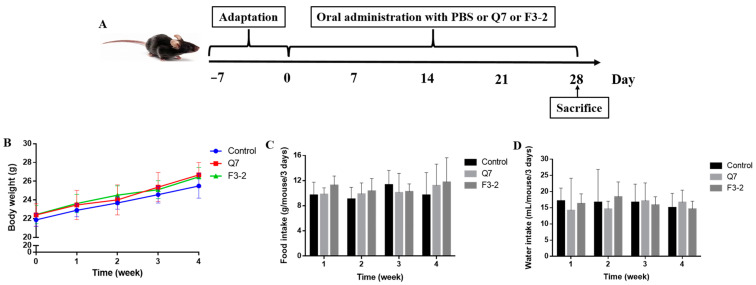
Animal experiment design (**A**) and the changes in body weight (**B**), food intake (**C**) and water intake (**D**) of mice gavaged with *L. plantarum* Q7 and *L. plantarum* F3-2.

The procedure of the animal experiment was indicated in Figure 1A. When *L. plantarum* Q7 and *L. plantarum* F3-2 were orally administered to mice, the changes in the body weight, food and water intake of mice were monitored, and the results were shown in Figure 1B–D. It was found that compared to mice fed with PBS, no significant differences were found in weekly body weight in the Q7 and F3-2 groups (*p* > 0.05). The intragastric administration of the two strains did not lead to any changes in the food and water consumption of mice (*p* > 0.05).

### 3.2. Effects of Bacteriocin-Producing L. plantarum on Intestinal Chemical Barrier of Mice

**Figure 2:**
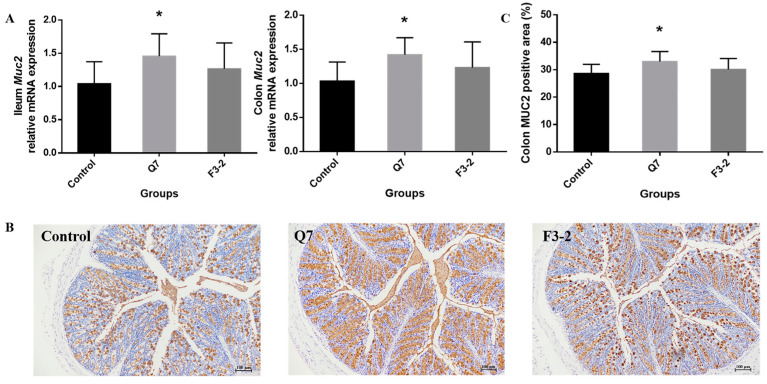
Effects of *L. plantarum* Q7 and *L. plantarum* F3-2 on mucin *Muc2* gene expression in ileum and colon (**A**), and immunohistochemistry, (**B**) as well as positive area of MUC2 in colon (**C**) of mice. Scale bar: 100 μm. * *p* < 0.05: strain groups compared with control group.

To explore the effects of two strains of *L. plantarum* on the intestinal chemical barrier, intestinal mucin MUC2 expression in mice was measured. It was illustrated in Figure 2A that the expression levels of *Muc2* in the ileum and colon were up-regulated remarkably with the oral administration of *L. plantarum* Q7 (*p* < 0.05), while *L. plantarum* F3-2 intervention had no evident effect on *Muc2* (*p* > 0.05). Further analysis was conducted via immunohistochemistry. After the mice were gavaged with *L. plantarum* Q7, the positive area of MUC2 in colon was increased significantly (*p* < 0.05), while there was no significant difference between the F3-2 group and the control group (*p* > 0.05), which was in line with the result of *Muc2* gene expression (Figure 2B,C).

### 3.3. Effects of Bacteriocin-Producing L. plantarum on Intestinal Physical Barrier of Mice

**Figure 3:**
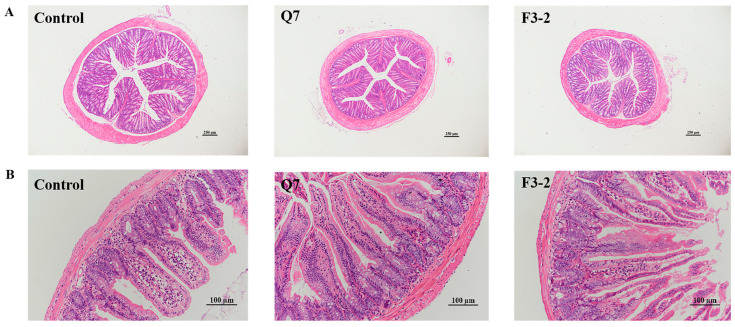
Effects of *L. plantarum* Q7 and *L. plantarum* F3-2 on histomorphology of colon (**A**) and ileum (**B**) in mice.

**Figure 4:**
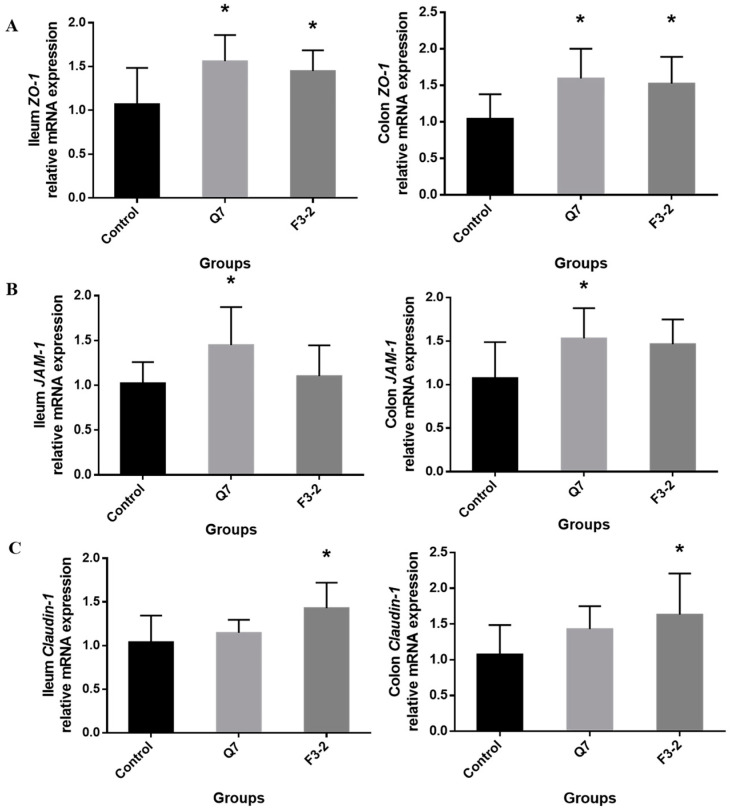
Effects of *L. plantarum* Q7 and *L. plantarum* F3-2 on gene expression of tight junction proteins *ZO-1* (**A**), *JAM-1* (**B**) and *Claudin-1* (**C**) in ileum and colon of mice. * *p* < 0.05: strain groups compared with control group.

The effects of two strains of *L. plantarum* on the intestinal physical barrier were evaluated via observation of the intestinal tissue morphology and the detection of tight junction proteins in mice. It was observed that no pathologic alterations in the colonic tissue were found in groups that received two strains relative to the control group (Figure 3A). The villus height and the ratio of villus height to crypt depth in ileum were significantly increased (*p* < 0.05), and the crypt depth remained almost unchanged (*p* > 0.05) when mice were fed with *L. plantarum* Q7 and *L. plantarum* F3-2 (Figure 3B and Table 2). There was no significant difference in the ileal tissue of mice between the two strain-treated groups (*p* > 0.05). The gene expression levels of the tight junction proteins *ZO-1*, *JAM-1* and *Claudin-1* in the ileum and colon were measured. Figure 4 indicated that *L. plantarum* Q7 treatment significantly up-regulated the gene expression of *ZO-1* and *JAM-1* (*p* < 0.05), and *L. plantarum* F3-2 treatment significantly up-regulated *ZO-1* and *Claudin-1* (*p* < 0.05).

### 3.4. Effects of Bacteriocin-Producing L. plantarum on Intestinal Immunological Barrier of Mice

**Figure 5:**
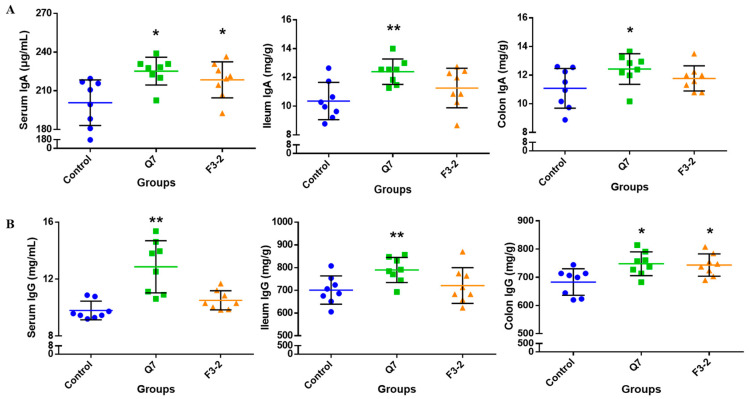
Effects of *L. plantarum* Q7 and *L. plantarum* F3-2 on the contents of immunoglobulins IgA (**A**) and IgG (**B**) in serum, ileum and colon of mice. * *p* < 0.05: strain groups compared with control group. ** *p* < 0.01: strain groups compared with control group.

**Figure 6:**
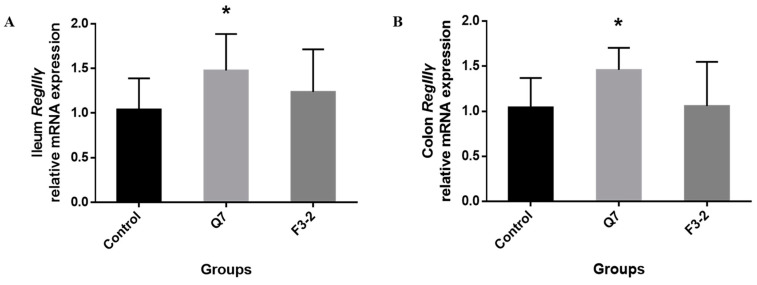
Effects of *L. plantarum* Q7 and *L. plantarum* F3-2 on gene expression of endogenous antimicrobial peptide *RegIIIγ* in ileum (**A**) and colon (**B**) of mice. * *p* < 0.05: strain groups compared with control group.

**Figure 7:**
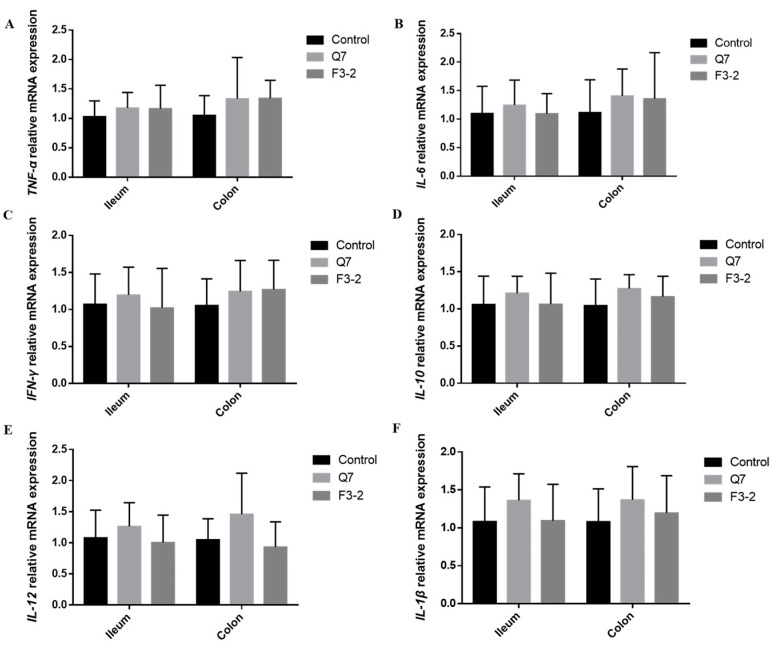
Effects of *L. plantarum* Q7 and *L. plantarum* F3-2 on gene expression of inflammatory cytokines *TNF-α* (**A**), *IL-6* (**B**), *IFN-γ* (**C**), *IL-10* (**D)**, *IL-12* (**E**) and *IL-1β* (**F**) in ileum and colon of mice.

The determination of immunoglobulins, endogenous antibacterial peptide and inflammatory cytokines in the intestine of mice was carried out to assess the roles of the two *L. plantarum* strains in the intestinal immunological barrier. It can be seen from Figure 5 that the administration of two strains caused a marked increase in IgA content in the serum of mice (*p* < 0.05). The contents of IgA in the ileum (*p* < 0.01) and colon (*p* < 0.05) were remarkably up-regulated in the Q7 group, but no obvious change occurred in the IgA concentration in mice intestine in the F3-2 group (*p* > 0.05). Moreover, the IgG contents in the serum (*p* < 0.01), ileum (*p* < 0.01) and colon (*p* < 0.05) were significantly increased when *L. plantarum* Q7 was administered, while *L. plantarum* F3-2 intervention only remarkably increased IgG content in the colon of mice (*p* < 0.05). The gene expression of *RegIIIγ* was shown in Figure 6. It was found that in comparison with the control group, there was evident up-regulation of *RegIIIγ* gene expression in the ileum and colon of mice gavaged with *L. plantarum* Q7 (*p* < 0.05), but *L. plantarum* F3-2 had no significant influence on *RegIIIγ* (*p* > 0.05). Furthermore, the gene expression levels of six inflammatory cytokines in the ileum and colon were detected. Figure 7 illustrated that no apparent differences were found in the gene expression of *TNF-α*, *IL-6*, *IFN-γ*, *IL-10*, *IL-12* and *IL-1β* among the three groups (*p* > 0.05). However, it was worth mentioning that the gene expression of the above inflammatory cytokines showed an upward trend in the Q7 group compared with that of the control group.

### 3.5. Effects of Bacteriocin-Producing L. plantarum on Intestinal Biological Barrier of Mice

**Figure 8:**
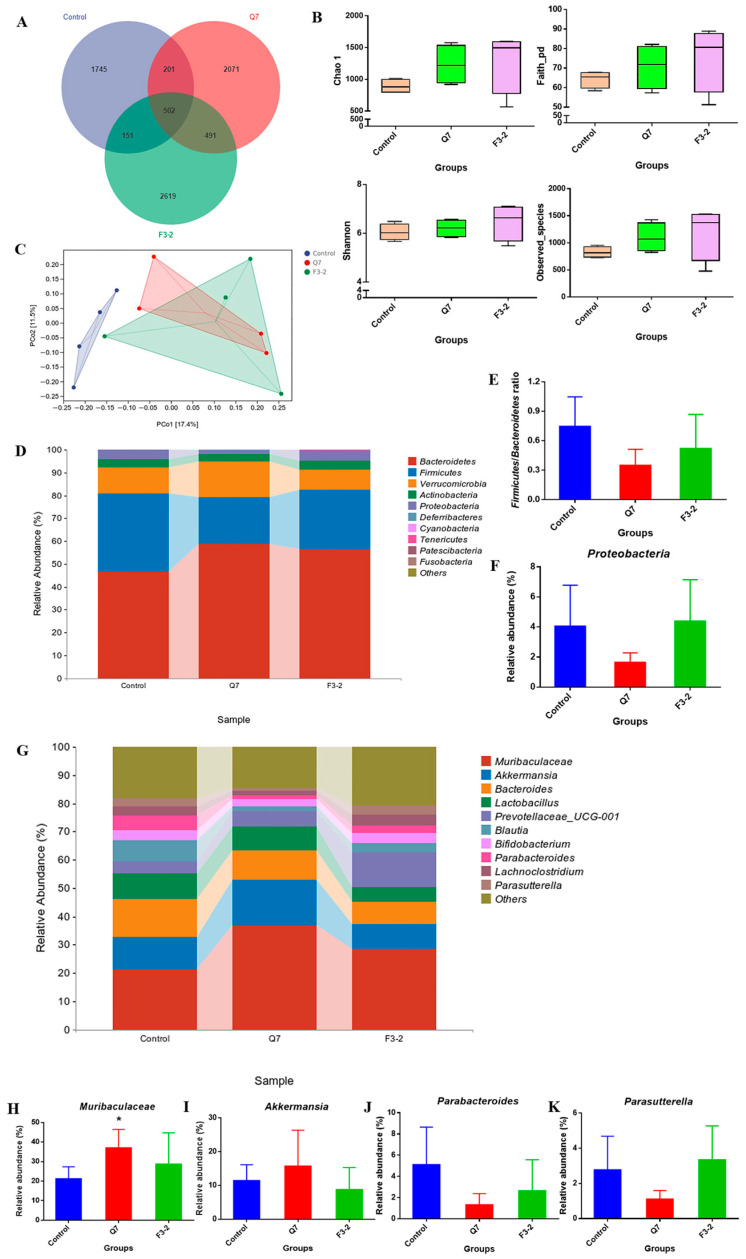
Effects of *L. plantarum* Q7 and *L. plantarum* F3-2 on gut microbiota of mice. (**A**) Venn diagram of ASVs/OUTs. (**B**) α-diversity indexes. (**C**) β-diversity evaluated via PCoA. (**D**) Bar graphs of bacteria relative abundance at phylum level. (**E**) The F/B ratio. (**F**) *Proteobacteria* relative abundance. (**G**) Bar graphs of bacteria relative abundance at genus level. (**H**) *Muribaculaceae* relative abundance. (**I**) *Akkermansia* relative abundance. (**J**) *Parabacteroides* relative abundance. (**K**) *Parasutterella* relative abundance. * *p* < 0.05: strain groups compared with control group.

The fecal microbiota of mice was analyzed to investigate the effects of the two *L. plantarum* strains on the intestinal biological barrier. As shown in Figure 8A, 502 common OTUs were identified among three groups, and the OTUs in the Q7 and F3-2 groups were more abundant than that in the control group. When the α-diversity of the microbial communities in the three groups was comparatively studied, it turned out that α-diversity indexes such as Chao 1, Faith_pd, Shannon and Observed_species were increased slightly after mice were gavaged with the two strains, but there was no evident difference (*p* > 0.05) (Figure 8B). PCoA was used to evaluate the β-diversity of the intestinal microbiota, and it was observed that the microbiota of the two strain-treated groups were separated from the control group (Figure 8C), indicating that there were changes in the composition and structure of the gut bacteria to some extent due to the treatment with the two strains. Therefore, the species composition of intestinal microbiota in mice was further analyzed. As evident from Figure 8D, *Bacteroidetes* was the most dominant phylum in the microbial communities among three groups, followed by *Firmicutes* and *Verrucomicrobia*. After the intervention of the two strains, the ratio of *Firmicutes* to *Bacteroidetes* (F/B) in the feces of mice showed a decreasing trend (*p* > 0.05) (Figure 8E). In the control group, the relative abundance of *Proteobacteria* was 4.04%, which was reduced to 1.64% after the intragastric administration of *L. plantarum* Q7 (Figure 8F). The difference in the gut microbiota at the genus level was revealed in Figure 8G, and suggested that when *L. plantarum* Q7 was administered, the relative abundance of the beneficial bacteria *Muribaculaceae* was increased markedly (*p* < 0.05), and *Muribaculaceae* abundance showed an upward trend with *L. plantarum* F3-2 intervention (*p* > 0.05) (Figure 8H). Furthermore, the abundance of *Akkermansia* in intestinal microorganisms was increased from 11.43% to 15.72% in mice treated with *L. plantarum* Q7 (Figure 8I). In contrast, the relative abundance of *Parabacteroides* was declined from 5.10% in the control group to 1.31% and 2.63% in the Q7 and F3-2 groups, respectively (Figure 8J). *Parasutterella* is common opportunistic pathogen, and its abundance in the control group was 2.76%, which was decreased to 1.10% with *L. plantarum* Q7 supplementation (Figure 8K). The effects of the two strains on SCFAs, the main metabolites of the intestinal microbiota, were also studied. The data in Table 3 demonstrated that the contents of acetic acid (*p* < 0.05), propionic acid (*p* < 0.05) and total SCFAs (*p* < 0.01) in the Q7 group were significantly higher than those in the control group. After mice received *L. plantarum* F3-2 via oral gavage, the concentration of acetic acid showed a rising trend (*p* > 0.05), and the content of total SCFAs was increased significantly compared to the control group (*p* < 0.05).

## 4. Discussion

A large number of bacteriocins from lactic acid bacteria have been identified, and they are mainly used as biological preservatives in food preservation studies and to alleviate pathogens infection in host [20]. With the wide application of bacteriocins, a growing number of studies have confirmed that bacteriocins synthesized by probiotics are beneficial for regulating the immune system, modulating the intestinal microbiota structure and alleviating the inflammation response [14]. Dabour et al. [21] found that the oral administration of pediocin PA-1 from *Pediococcus acidilactici* UL5 to mice reduced the viable counts of the pathogen *Listeria monocytogenes* in the intestine, which would basically not perturb the gut microbial equilibrium. The bacteriocin-producing *L. plantarum* NCIMB8826 has been shown to reduce the levels of pro-inflammatory cytokines TNF-α and IL-6 in IBD mice [22]. In view of the above reports, bacteriocin-producing probiotics displayed beneficial effects on host intestinal health, but research on their impacts on the intestinal barrier is not clear enough. Therefore, in this study, bacteriocin-producing *L. plantarum* Q7 and *L. plantarum* F3-2 with probiotic potential were given to mice via intragastric administration, and it was found that the two strains did not affect the body weight and basic physiological activity of mice, such as feeding and drinking, so they were preliminarily regarded as safe organisms, and their effects on the intestinal barrier of mice were further investigated.

The intestinal chemical barrier is mainly composed of the mucus layer. MUC2 is the main component of the gut mucus layer, and covers the top of the intestinal epithelium to play a crucial role in enhancing mucosal immunoregulatory and maintaining intestinal homeostasis [23]. *L. plantarum* Q7 promoted the expression of mucin MUC2, which was conductive to lubricating the intestinal tract, providing adhesion sites for the gut symbiotic microbiota, and resisting pathogens invasion, thereby preventing inflammation and damage, and protecting the intestinal chemical barrier. However, *L. plantarum* F3-2 had no significant influence on MUC2, which revealed differences in the effects of various bacteriocin-producing strains on the intestinal chemical barrier. In terms of the intestinal physical barrier, the two strains were helpful in maintaining good colonic tissue morphology in mice, and improving the ratio of villus height to crypt depth of the ileum, which enhanced the intestinal absorption of nutrients. Tight junction proteins are the most vital structures of the intestinal physical barrier. The gene expression of *ZO-1* and *JAM-1* in the intestine of mice were remarkably up-regulated with *L. plantarum* Q7 intervention, and *ZO-1* and *Claudin-1* showed a significant increase after *L. plantarum* F3-2 supplementation, indicating that bacteriocin-producing *L. plantarum* could protect the intestinal physical barrier by promoting tight junction proteins expression, which was in accordance with the study of Heeney et al. [24].

When the effects of bacteriocin-producing *L. plantarum* on the intestinal immunological barrier were explored, the results revealed that the two strains promoted the release of IgA and IgG in the serum and intestine to varying degrees, which suggested that bacteriocin-producing strains could strengthen the intestinal immune function, but there existed differences when different strains acted on immunoglobulins in distinct tissues of mice. Antimicrobial peptides are crucial participants in host natural defense and play a significant part in innate immunity [25]. The antimicrobial peptide RegIIIγ, produced by Paneth cells, can protect mice from pathogens infection, regulate intestinal microecological balance, and maintain immune homeostasis [26]. *L. plantarum* Q7 remarkably up-regulated the gene expression of *RegIIIγ* in the ileum and colon of mice to enhance the antibacterial, anti-inflammatory and immunomodulatory functions of the intestine, which demonstrated that the intervention of exogenous bacteriocin-producing strains could affect the immune function of the host by regulating the expression of endogenous antimicrobial peptides. In addition to immunoglobulins and antimicrobial peptides, inflammatory cytokines are also closely related to immune function. With the increasing studies on bacteriocins regulating cytokines secretion, bacteriocins have been confirmed to promote the production of inflammatory cytokines to enhance body immunity, and decrease proinflammatory cytokine levels to develop anti-inflammatory function [14]. Małaczewska et al. [27] found that Nisin stimulated IL-1β and IL-6 levels in peripheral blood mononuclear cells (PBMC), and increased the CD4^+^ CD8^+^ T cells proportion, but when PBMC was stimulated by lipopolysaccharide (LPS), Nisin reduced the production of LPS-induced proinflammatory factor IL-6, reflecting the dual roles of Nisin in immunomodulation and anti-inflammation. In our study, the two strains had little effect on intestinal inflammatory cytokines in mice, but the gene expression of inflammatory cytokines showed an upward trend after the oral administration of *L. plantarum* Q7. Cui et al. [28] reported that bacteriocin-producing *Pediococcus pentosaceus* treatment in the short term promoted an increase in inflammatory cytokines in the colon of healthy mice, which initiated the immune response and activated the immune regulation system. Similarly, the up-regulation trend of *L. plantarum* Q7 on inflammatory cytokines reflected its potential to enhance body immunity and treat immunodeficiency. However, it is necessary to further analyze the anti-inflammatory activity of strains in a state of inflammation, so as to evaluate the immunomodulatory function of strains comprehensively. The two bacteriocin-producing strains indicated strain specificity in terms of their influences on intestinal immunity. In particular, *L. plantarum* Q7 increased the contents of immunoglobulins and the expression level of antimicrobial peptide without causing an inflammatory response, which was a critical way to protect the intestinal immunological barrier in mice.

The intestinal biological barrier mainly consists of gut microorganisms. The oral delivery of two strains led to a slight increase in Chao 1, Faith_pd, Shannon and Observed_species, suggesting that these strains slightly increased the richness, uniformity and diversity of the gut microbiota with no significant difference, which was slightly differentiated from previous research. Qiao et al. [29] found that *P. acidilactici* with bacteriocin production ability could significantly enhance the Chao 1 index of the intestinal microbiota in mice, but the Shannon index showed an insignificant downward trend. Hence, it can be concluded that different bacteriocin-producing lactic acid bacteria had various effects on the α-diversity of intestinal communities.

According to the results of β-diversity, *L. plantarum* Q7 and *L. plantarum* F3-2 had certain impacts on the composition and structure of the intestinal microbiota. After the intervention of the two strains, F/B in the intestine of mice tended to be decreased. It was reported that the intake of *Lactobacillus* could help reduce F/B, thereby inhibiting the occurrence of metabolic diseases [30], which indicated that the two strains had the potential to regulate the intestinal microbiota structure and ameliorate metabolic disorders. *Proteobacteria* is regarded as a signature of dysbiosis and disease risk [31]. *L. plantarum* Q7 reduced *Proteobacteria* abundance to inhibit intestinal pathogens growth, which might be attributed to the production of bacteriocin from *L. plantarum* Q7. *Muribaculaceae* and *Akkermansia* are beneficial bacteria in mice feces, and were up-regulated in the Q7 group. The regulation of the abundance of *Muribaculaceae* and *Akkermansia* could provide a basis for ameliorating inflammation and metabolic diseases such as obesity [32]. Similarly, it was found that bacteriocin-producing *Lactiplantibacillus acidophilus* JCM 1132 significantly increased *Akkermansia* abundance in the intestinal tract of mice after a one-week withdrawal period, and it was speculated that bacteriocin might have induced the change of *Akkermansia* by killing specific bacteria [33]. Moreover, it is confirmed that *Akkermansia* can degrade mucin specifically to stimulate the secretion of new mucin and promote MUC2 expression, which is essential for accelerating intestinal epithelial regeneration and repairing damaged intestinal mucosa [34]. In our study, the gene expression of *Muc2* was markedly up-regulated in the Q7 group, which might be associated with the increasing abundance of *Akkermansia* in the intestinal microbiota due to *L. plantarum* Q7 treatment. *Parabacteroides* and *Parasutterella* are potentially harmful bacteria. An increase in *Parabacteroides* abundance can exacerbate infection and increase the permeability of the intestine [35]. An increase in *Parasutterella* will bring about a decrease in gut microbiota diversity or microecological dysbiosis, which is associated with the occurrence of irritable bowel syndrome, obesity and type II diabetes [36]. The two strains reduced the abundance of harmful bacteria, which was beneficial to improve obesity and chronic intestinal inflammation and maintain intestinal homeostasis. Our findings reflected that the two *L. plantarum* strains were conductive to improving the structure of the intestinal microbiota in healthy mice, but there also existed certain differences in the regulation effects of different strains, which was also proposed by Qiao et al. [29] previously. At present, the research on the modulation of gut microbiota structure through bacteriocins and bacteriocin-producing probiotics has become a hotspot. Riboulet-Bisson et al. [37] pointed out that the bacteriocin-producing *Lactiplantibacillus salivarius* UCC118 had a subtle influence on the mice microbiota via a partial bacteriocin-dependent mechanism. Gebhart et al. [38] obtained a modified bacteriocin, which could prevent *Clostridium difficile* from colonizing in the host intestine without disrupting protective indigenous microbiota. The above studies showed that bacteriocins and bacteriocin-producing probiotics promoted subtle but favorable changes in intestinal communities structure without destroying the original protective microbiota, which was in line with our study.

As the main energy source of the intestinal epithelium, SCFAs play a critical role in protecting the intestinal barrier. The two strains increased the contents of SCFAs, which contributed to regulating host immunity, inflammatory response and energy metabolism. Smith et al. [39] reported that *Muribaculaceae* abundance was correlated with propionic acid. It was demonstrated that *Parasutterella* abundance was markedly lower based on the accumulation of total SCFAs in the feces of beagle dogs [40]. Therefore, there was a correlation between intestinal microbes and SCFAs, and it could be inferred that *L. plantarum* Q7 and *L. plantarum* F3-2 regulated the gut microbiota to affect the production of SCFAs, thus protecting the intestinal biological barrier.

## 5. Conclusions

In conclusion, our study demonstrated that *L. plantarum* Q7 and *L. plantarum* F3-2 could protect the intestinal chemical, physical, immunological and biological barriers of healthy mice at different degrees. The effects of *L. plantarum* Q7 were better than those of *L. plantarum* F3-2 regarding the levels of mucin, immunoglobulins, antibacterial peptide and SCFAs. To fully evaluate the impacts and mechanisms of bacteriocin-producing probiotics on the intestinal barrier, further research is required to explore the injury repair effects of *L. plantarum* Q7 and its mutant strain with knock-out of the gene related to bacteriocin production on the intestinal barrier of mice with intestinal dysfunction. These findings would provide a reference for the development of different bacteriocin-producing probiotics with beneficial characteristics to strengthen intestinal barrier function.

## Figures and Tables

**Table 1:** Primers used in RT-qPCR.

Genes	Forward Sequences (5′-3′)	Reverse Sequences (5′-3′)
*β-actin*	F: GTGCTATGTTGCTCTAGACTTCG	R: ATGCCACAGGATTCCATACC
*Muc2*	F: TGCTGACGAGTGGTTGGTGAATG	R: TGATGAGGTGGCAGACAGGAGAC
*ZO-1*	F: GCTGCCTCGAACCTCTACTC	R: TTGCTCATAACTTCGCGGGT
*JAM-1*	F: AGTTCGTCCAAGGCAGCACAAC	R: AGAAGGTGACTCGGTCCGCATAG
*Claudin-1*	F: GCTGGGTTTCATCCTGGCTTCTC	R: CCTGAGCGGTCACGATGTTGTC
*RegIIIγ*	F: GCTTCCTTCCTGTCCTCCATGATC	R: ATCACATCAGCATTGCTCCACTCC
*TNF-α*	F: GCGACGTGGAACTGGCAGAAG	R: GCCACAAGCAGGAATGAGAAGAGG
*IL-6*	F: ACTTCCATCCAGTTGCCTTCTTGG	R: TTAAGCCTCCGACTTGTGAAGTGG
*IFN-γ*	F: CTGGAGGAACTGGCAAAAGGATGG	R: GACGCTTATGTTGTTGCTGATGGC
*IL-10*	F: GAGGATCAGCAGGGGCCAGTAC	R: AAGGCAGTCCGCAGCTCTAGG
*IL-12*	F: TCTTTGATGATGACCCTGTGCCTTG	R: GTGATTCTGAAGTGCTGCGTTGATG
*IL-1β*	F: TCGCAGCAGCACATCAACAAGAG	R: TGCTCATGTCCTCATCCTGGAAGG

**Table 2:** Effects of *L. plantarum* Q7 and *L. plantarum* F3-2 on villus height and crypt depth of ileum in mice.

Groups	Villus Height (μm)	Crypt Depth (μm)	Villus Height/Crypt Depth
Control	247.57 ± 19.51 ^b^	87.53 ± 14.44 ^a^	2.90 ± 0.53 ^b^
Q7	344.82 ± 20.27 ^a^	84.41 ± 10.33 ^a^	4.14 ± 0.58 ^a^
F3-2	337.36 ± 22.82 ^a^	85.01 ± 9.94 ^a^	4.02 ± 0.56 ^a^

Different letters in the same column indicate significant differences (*p* < 0.05).

**Table 3:** Effects of *L. plantarum* Q7 and *L. plantarum* F3-2 on the contents of acetic acid, propionic acid, butyric acid and total SCFAs in feces of mice.

Groups	Acetic Acid (μg/g)	Propionic Acid (μg/g)	Butyric Acid (μg/g)	Total SCFAs (μg/g)
Control	378.59 ± 83.50	242.93 ± 49.57	189.53 ± 39.31	811.04 ± 48.25
Q7	531.15 ± 90.89 *	320.38 ± 63.08 *	200.78 ± 49.40	1052.31 ± 139.84 **
F3-2	480.68 ± 121.61	257.81 ± 63.02	208.98 ± 35.16	947.46 ± 135.75 *

* *p* < 0.05: strain groups compared with control group in the same column. ** *p* < 0.01: strain groups compared with control group in the same column.

## Data Availability

The data presented in this study are available on request from the corresponding author.
